# Burden, trends, and predictions of liver cancer in China, Japan, and South Korea: analysis based on the Global Burden of Disease Study 2021

**DOI:** 10.1007/s12072-024-10763-6

**Published:** 2025-01-11

**Authors:** Si Yang, Yujiao Deng, Yi Zheng, Jing Zhang, Dongdong He, Zhijun Dai, Changcun Guo

**Affiliations:** 1 Department of Digestive Diseases, Xijing Hospital, Air Force Medical University, Xi’an, 710000 Shaanxi China; 2 Department of Digestive Diseases, The Second Affiliated Hospital of Xi’an Jiaotong University, Xi’an, China; 3 Department of Oncology, Xijing Hospital, Air Force Medical University, Xi’an, China; 4 Department of Breast Surgery, The First Affiliated Hospital, College of Medicine, Zhejiang University, Hangzhou, 310003 Zhejiang China

**Keywords:** Liver cancer, Incidence, Death, Disability-adjusted life years, China, Japan, South Korea, Risk factor, Etiologies

## Abstract

**Background:**

Liver cancer (LC) is a major concern in the Asia-Pacific region, particularly in China, Korea, and Japan. In this study, we aimed to investigate the burden, trends, and predictions related to LC in these countries.

**Methods:**

Using data from the Global Burden of Disease Study 2021, the epidemiological characteristics [incidence, deaths, and disability-adjusted life-years (DALYs)] for LC were analysed and stratified by specific etiologies in China, Japan, and South Korea. We examined temporal trends in LC burden over the last 32 years and projected changes over the following 25 years. The risk factors associated with LC deaths and DALYs were also investigated.

**Results:**

In 2021, the highest LC-related incidence, mortality, and DALYs were recorded in China (196,637 incidents, 172,068 mortalities, and 4,890,023 DALYs), and the lowest in South Korea (18,642 incidents, 13,674 deaths, and 326,336 DALYs). South Korea recorded the highest age-standardized rates (ASRs) of incidence, mortality, and DALYs for LC (19.94 per 100,000, 14.53 per 100,000, and 354.57 per 100,000), and Japan the lowest (9.89, 7.29, and 145.74, respectively). From 1990 to 2021, LC incidents and deaths in the three countries increased, and the trends in ASRs decreased. LC incidents and deaths caused by five etiologies also increased in the past 32 years, and non-alcoholic steatohepatitis (NASH) was the largest increasing etiologies in China. Infections with hepatitis B virus remained the leading cause of LC in China and South Korea, while hepatitis C virus was the prevailing cause in Japan. High body mass index (BMI) was the most sharply increasing risk factor in China. Alcohol and drug use were the main risk factors for LC in South Korea and Japan, respectively. The LC burden in the three countries was projected to rise steadily between 2022 and 2046.

**Conclusions:**

LC remains a significant disease burden in China, Japan, and South Korea now and over the next 25 years. Regarding etiologies and risk factors, NASH and high BMI in China, alcohol use in South Korea, and drug use in Japan should receive significant attention.

**Supplementary Information:**

The online version contains supplementary material available at 10.1007/s12072-024-10763-6.

## Introduction

Liver cancer (LC) is the sixth most diagnosed cancer and the third leading cause of cancer-related deaths worldwide, with 865,269 new cases and 757,948 deaths in 2022 according to GLOBOCAN 2022 [1]. The disease is complicated, with several probable etiologies, and is associated with various risk factors [2]. The major etiologies of LC include infection with hepatitis B or C virus (HBV or HCV, respectively), alcohol use, and non-alcoholic steatohepatitis (NASH) [3]. Additional risk factors for developing LC include aflatoxin intake, diabetes, obesity, excessive alcohol consumption, and tobacco use [4–6]. Notably, the last 10 years have seen changes in the burden, etiologies, and risk factors of LC. LC is particularly common in the Asia-Pacific region, and its epidemiology in this region has been changing. The Asia-Pacific area contributes to nearly 75% of all LC incidences [7] and three-quarters of global deaths due to LC [8]. China, Japan, and South Korea account for one-fifth of the global population and one-fourth of the world's gross domestic product, making them the most important economic engines in the Asia-Pacific region. Therefore, it is of paramount importance that healthcare professionals in China, Korea, and Japan document the burden of LC in the region and examine new strategies to counteract this highly prevalent disease. China, Japan, and South Korea exhibit similar ethnic, ancestral, and cultural histories, yet they differ greatly in terms of population size and composition as well as degree of socioeconomic development. Although a great deal of effort has been made to prevent and treat LC in these countries, varying levels of achievements have been reported owing to differing socioeconomic situations and prevention and control approaches. Identifying variations in the burden, patterns, and risk factors of LC among these three nations can help monitor the success of national screening initiatives and allow knowledge transfer from each of the national tumor-prevention strategies. In this study, we aimed to describe the burden, patterns, and risk factors of LC in these three nations from 1990 to 2021 and predict trends in disease burden for the next 25 years. Our study provides epidemiological information for the effective prevention and control of LC in these countries.

## Methods

### Data source

The Global Burden of Disease (GBD) Study 2021 [9] provided us with data regarding new LC cases, deaths, disability-adjusted life-years (DALYs), and age-standardized rates (ASRs) for LC incidence, deaths, and DALYs based on differences in sex, age groups, and etiologies from 1990 to 2021. The data were downloaded from the Global Health Data Exchange (GHDx) query tool (https://vizhub.healthdata.org/gbd-results/). In this tool, we selected “Cause of death or injury” as the “GBD Estimate”; “Liver cancer”, “Liver cancer due to hepatitis B”, “Liver cancer due to hepatitis C”, “Liver cancer due to alcohol use”, “Liver cancer due to other causes”, and “Liver cancer due to NASH” as the “Cause”; “Incidence”, “Deaths”, and “DALYs” as the “Measure”; “Number” and “Rate” as the “Metric”; and “Global”, “East Asia & Pacific-World Bank [WB]”, “Europe & Central Asia -WB”, “Latin America & Caribbean -WB”, “Middle East & North Africa- WB”, “North America”, “South Asia-WB”, “Sub-Saharan Africa -WB”, “China”, “Japan”, and “Republic of Korea” as the “Location”. Risk factors associated with LC mortality and DALYs in China, Japan, and South Korea were analysed based on the GBD Study 2021. The risk factor hierarchy was previously described in GBD 2019 [10] and divided into four levels, ranging from the widest (level 1) to the most specific (level 4). The level 1 group of risk factors includes behavioural, environmental and occupational, and metabolic risks. In this study, we assessed the level 2 group of risks factors. Specifically, in the GHDx query tool, we selected “Risk factor” as the “GBD Estimate”; “Deaths” and “DALYs” as the “Measure”; “Number”, “Percent”, and “Rate” as the “Metric”; “Select only level 2 risks” as the “Risk”; “Liver cancer” as the “Cause”; and “China”, “East Asia & Pacific-WB”, “Global”, “Japan”, and “Republic of Korea” as the “Location”.

### Statistical analyses

Incidence, deaths, DALYs, and their rates were reported with 95% uncertainty intervals (UIs). The methods for LC burden estimation have been reported previously [11]. The GBD collaborators classify the etiologies for LC into five groups: hepatitis B, hepatitis C, alcohol use, NASH, and other causes. “Cryptogenic” or “idiopathic” LC, LC caused by hemochromatosis, autoimmune hepatitis, and Wilson’s disease were included in the “other causes” category. The decomposition analysis illustrated how the population, age, and epidemiological changes contributed to the varying LC burden between 1990 and 2021 [12]. The Joinpoint software (version 4.9.0) was used to calculate and analyse the annual percentage change (APC) and average annual percentage change (AAPC) in ASR for LC incidence, deaths, and DALYs from 1990 to 2021 using joinpoint regression analysis [13]. To forecast the number and ASR of LC cases from 2021 to 2046, we used a log-linear age–time–cohort model and the Nordpred package [14]. All statistical analyses were performed using the R software (version 4.4.0), and statistical significance was set as a *p* value of < 0.05.

## Results

### LC burden in the world and in seven regions

Table 1 provides an overview of the global distribution of LC incidents, deaths, and DALYs in 2021. Globally, there were 529,202 cases (95% UI 480,339–593,849), 483,875 (440,400–540,177) deaths, and 12,887,652 (11,673,533–14,472,228) DALYs. There were 310,535 newly reported LC cases in East Asia & Pacific, accounting for 58.74% of the new global LC cases. Subsequent continental distributions delineated Europe & Central Asia, South Asia, Sub-Saharan Africa, North America, Middle East & North Africa, and Latin America & Caribbean contributing to 12.84%, 8.17%, 6.98%, 6.61%, 3.70%, and 2.96%, respectively. East Asia & Pacific accounted for 56.26% of global deaths due to LC, followed by Europe & Central Asia (13.39%), South Asia (9.22%), Sub-Saharan Africa (7.78%), North America (5.79%), Middle East & North Africa (4.12%), and Latin America & Caribbean (3.44%). East Asia & Pacific had the highest age-standardized incidence rate (ASIR) and age-standardized death rate (ASDR) (9.55 and 8.37 per 100,000, respectively), followed by Sub-Saharan Africa (6.82 and 7.33 per 100,000) and Middle East & North Africa (5.61 and 5.89 per 100,000).

**Table 1:** The ASR and number of liver cancer cases in the world and in seven regions in 1990 and 2021

Variables	World	East Asia & Pacific	Europe & Central Asia	Latin America & Caribbean	Middle East & North Africa	North America	South Asia	Sub-Saharan Africa
*1990*								
Incidence rate (per 100,000)	5.90 (5.43–6.48)	10.90 (9.79–12.05)	3.15 (3.03–3.25)	1.99 (1.91–2.08)	5.24 (4.01–7.49)	2.34 (2.23–2.40)	2.15 (1.92–2.40)	8.38 (5.84–11.83)
Incidence numbers	244,689 (224,795–268,549)	155,935 (139,022–172,971)	33,364 (32,193–34,432)	5489 (5283–5719)	6764 (5277–9460)	8002 (7610–8220)	14,194 (12,906–15,653)	20,617 (14,436–28,608)
Death rate (per 100,000)	5.86 (5.38–6.46)	10.73 (9.63–11.86)	3.19 (3.07–3.30)	2.14 (2.04–2.24)	5.6 (4.27–8.06)	2.00 (1.90–2.06)	2.25 (2.01–2.52)	8.92 (6.23–12.63)
Death numbers	238,969 (218,717–263,037)	149,861 (133,608–166,093)	34,029 (32,796–35,181)	5716 (5492–5966)	6920 (5351–9777)	7009 (6622–7218)	14,242 (12,877–15,755)	20,851 (14,587–29,131)
DALY rate (per 100,000)	172.86 (157.84–190.16)	320.95 (283.81–357.45)	82.37 (79.95–84.72)	55.69 (53.77–57.97)	146.87 (114.00–206.98)	51.89 (50.12–52.96)	65.00 (58.98–71.80)	246.83 (171.87–345.86)
DALY numbers	7,553,667 (6,897,511–8,296,182)	4,937,870 (4,344,078–5,509,801)	851,676 (825,355–876,920)	168,998 (163,462–175,676)	216,035 (172,722–294,926)	171,166 (164,893–174,975)	490,763 (446,162–539,078)	707,886 (499,189–969,083)
*2021*								
Incidence rate (per 100,000)	6.15 (5.58–6.90)	9.55 (8.23–11.21)	4.21 (3.95–4.42)	2.23 (2.06–2.39)	5.61 (4.67–6.52)	5.52 (5.15–5.74)	2.77 (2.51–3.05)	6.82 (5.71–8.26)
Incidence numbers	529,202 (480,339–593,849)	310,535 (267,660–365,279)	67,903 (63,248–71,304)	15,641 (14,448–16,786)	19,571 (16,224–22,974)	34,971 (32,428–36,412)	43,180 (38,936–47,608)	36,876 (30,331–45,448)
Death rate (per 100,000)	5.65 (5.13–6.30)	8.37 (7.20–9.84)	3.90 (3.64–4.10)	2.38 (2.20–2.55)	5.89 (4.90–6.83)	4.26 (3.96–4.43)	2.92 (2.64–3.22)	7.33 (6.14–8.90)
Death numbers	483,875 (440,400–540,177)	271,909 (234,210–319,507)	64,696 (59,933–68,034)	16,624 (15,362–17,800)	19,928 (16,495–23,386)	27,998 (25,830–29,194)	44,580 (40,403–49,235)	37,595 (30,994–46,425)
DALY rate (per 100,000)	149.29 (135.24–167.48)	226.36 (193.17–271.48)	93.09 (87.96–97.60)	58.51 (54.16–62.95)	149.28 (123.23–175.47)	106.02 (100.57–109.93)	79.79 (71.98–88.16)	193.80 (159.44–240.05)
DALY numbers	12,887,652 (11,673,533–14,472,228)	7,296,384 (6,219,804–8,758,337)	1,409,355 (1,331,543–1,477,830)	413,329 (383,044–444,334)	565,303 (466,514–665,120)	646,325 (610,317–670,644)	1,310,167 (1,181,492–1,451,121)	1,233,365 (997,279–1,553,525)
*1990–2021*								
ASIR (AAPC, 95% CI)	0.11 (0.06–0.16)	− 0.47 (− 0.52 to − 0.42)	0.92 (0.82–1.01)	0.32 (0.24–0.40)	0.24 (0.20–0.27)	2.82 (2.69–2.94)	0.83 (0.78–0.88)	− 0.67 (− 0.72 to − 0.62)
ASDR (AAPC, 95% CI)	− 0.11 (− 0.23 to 0.01)	− 0.74 (− 1.02 to − 0.46)	0.60 (0.41–0.80)	0.32 (− 0.06 to 0.70)	0.22 (− 0.12 to 0.56)	2.44 (2.13–2.76)	0.88 (0.58–1.19)	− 0.64 (− 0.71 to − 0.57)
ASR of DALY (AAPC, 95% CI)	− 0.46 (− 0.61 to − 0.31)	− 1.07 (− 1.46 to − 0.69)	0.34 (0.10–0.58)	0.16 (− 0.22 to 0.54)	0.09 (− 0.24 to 0.41)	2.32 (2.16–2.47)	0.68 (0.42–0.95)	− 0.79 (− 0.84 to − 0.73)

*ASIR* age-standardized incidence rate, *ASDR* age-standardized death rate, *ASR* age-standardized rate, *DALY* disability-adjusted life-year, *AAPC* average annual percentage change, *CI* confidence interval

Between 1990 and 2021, the ASIR, ASDR, and ASR of DALYs showed a decreasing trend in East Asia & Pacific and Sub-Saharan Africa (AAPC < 0, *p* < 0.05), but increasing trends in the other regions (AAPC > 0).

### LC burden in 2021 in China, Japan, and South Korea

In China, there were 196,637 incident cases (158,273–243,558), 172,068 deaths (139,621–212,496), and 4,890,023 (3,905,089–6,124,599) DALYs due to LC in 2021 (Table 2). The ASIR, ASDR, and ASR of DALYs for LC were 9.52 (7.72–11.78) per 100,000, 8.35(6.80–10.29) per 100,000, and 239.91 (191.98–299.37) per 100,000, respectively. In Japan, 39,163 (33,437–42,580) LC incident cases with an ASIR of 9.89 (8.84–10.57) were observed in 2021; LC caused 31,123 (26,459–33,773) deaths, with an ASDR of 7.29 (6.48–7.77). Moreover, LC resulted in 501,032 (445,194–534,862) DALYs with an ASR of 145.74 (133.93–153.13). In South Korea, there were 18,642 (15,182–22,884) incident cases, 13,674 (11,219–16,835) deaths, and 326,336 (267,089–403,348) DALYs in 2021. The ASIR, ASDR, and ASR of DALYs for LC were 19.94, 14.53, and 354.57, respectively.

**Table 2:** The ASR and number of liver cancer cases in the world, East Asia & Pacific, China, Japan, and South Korea from 1990 to 2021, both sexes

Variables	China			Japan			South Korea		
	1990	2021	Overall change, %	1990	2021	Overall change, %	1990	2021	Overall change, %
Incidence rate (per 100,000)	10.58 (8.94–12.43)	9.52 (7.72–11.78)	− 10.04 (− 32.28–17.51)	14.35 (13.62–14.76)	9.89 (8.84–10.57)	− 31.07 (− 36.00–− 27.17)	35.88 (27.14–45.09)	19.94 (16.27–24.49)	− 44.44 (− 58.65–− 24.12)
Incidence numbers	96,434 (80,971–113,769)	196,637 (158,273–243,558)	103.91 (52.37–168.23)	24,736 (23,506–25,434)	39,163 (33,437–42,580)	58.32 (41.55–69.77)	11,349 (8,533–14,319)	18,642 (15,182–22,884)	64.26 (20.97–127.12)
Death rate (per 100,000)	10.75 (9.12–12.61)	8.35 (6.80–10.29)	− 22.35 (− 41.21–0.71)	11.80 (11.16–12.12)	7.29 (6.48–7.77)	− 38.25 (− 42.92–− 35.26)	36.20 (27.45–45.34)	14.53 (11.97–17.79)	− 59.87 (− 70.09–− 45.46)
Death numbers	94,937 (79,884–111,527)	172,068 (139,621–212,496)	81.24 (36.07–137.11)	20,308 (19,265–20,828)	31,123 (26,459–33,773)	53.25 (35.74–63.70)	11,042 (8,305–13,956)	13,674 (11,219–16,835)	23.83 (− 8.58–71.08)
DALY rate (per 100,000)	334.52 (281.08–393.14)	239.91 (191.98–299.37)	− 28.28 (− 46.40–− 5.19)	307.72 (295.94–315.01)	145.74 (133.93–153.13)	− 52.64 (− 55.26–− 50.66)	986.11 (741.60–1,242.07)	354.57 (291.34–435.31)	− 64.04 (− 73.41–− 49.28)
DALY numbers	3,294,864 (2,763,029–3,879,589)	4,890,023 (3,905,089–6,124,599)	48.41 (10.75–96.81)	531,120 (511,158–543,692)	501,032 (445,194–534,862)	− 5.66 (− 14.03–− 0.48)	340,074 (252,485–430,554)	326,336 (267,089–403,348)	− 4.04 (− 30.11–35.45)

*ASR* age-standardized rate, *DALY* disability-adjusted life-year

The incidents, deaths, and DALYs of LC in China ranked first among the three nations and accounted for 63.32%, 63.28%, and 67.02%, respectively, of the LC burden in the East Asia & Pacific region and for 33.11%, 31.85%, and 37.94%, respectively, of the global LC burden. The ASIR, ASDR, and ASR of DALYs for LC in China were nearly equal to those for the East Asia & Pacific region and 1.47-fold that of the world (Table 2). The LC burden was the lowest in South Korea, whereas ASIR, ASDR, and ASR of DALYs ranked first among the three countries and at nearly 1.50-fold those of the East Asia & Pacific region and 2.30-fold those of the world.

Between 1990 and 2021, we discovered a rising trend in LC incidents and deaths globally, in the East Asia & Pacific region, and the three nations for both men and women (Table 2). The largest increases were observed in China (103.91% and 81.24%, respectively), and the lowest in Japan in terms of incident cases (58.32%) and in South Korea regarding deaths (23.83%).

To investigate the impact of ageing, population, and epidemiological changes on LC epidemiology, we conducted a decomposition analysis of the increasing number of cases. Between 1990 and 2021, ageing contributed to 93.2% of the increased incident cases of LC in China, and to 93.2% of the increased death cases from LC (Table S1). In Japan, ageing contributed to 80.5% of the increased incident cases of LC and to 87.1% of the increased death cases from LC (Table S1). In South Korea, ageing contributed to 129.4% of the increased incident cases of LC and to 124.0% of the increased death cases from LC (Table S1).

### Trends in ASIR, ASDR, and ASR of DALYs for LC

**Fig. 1:**
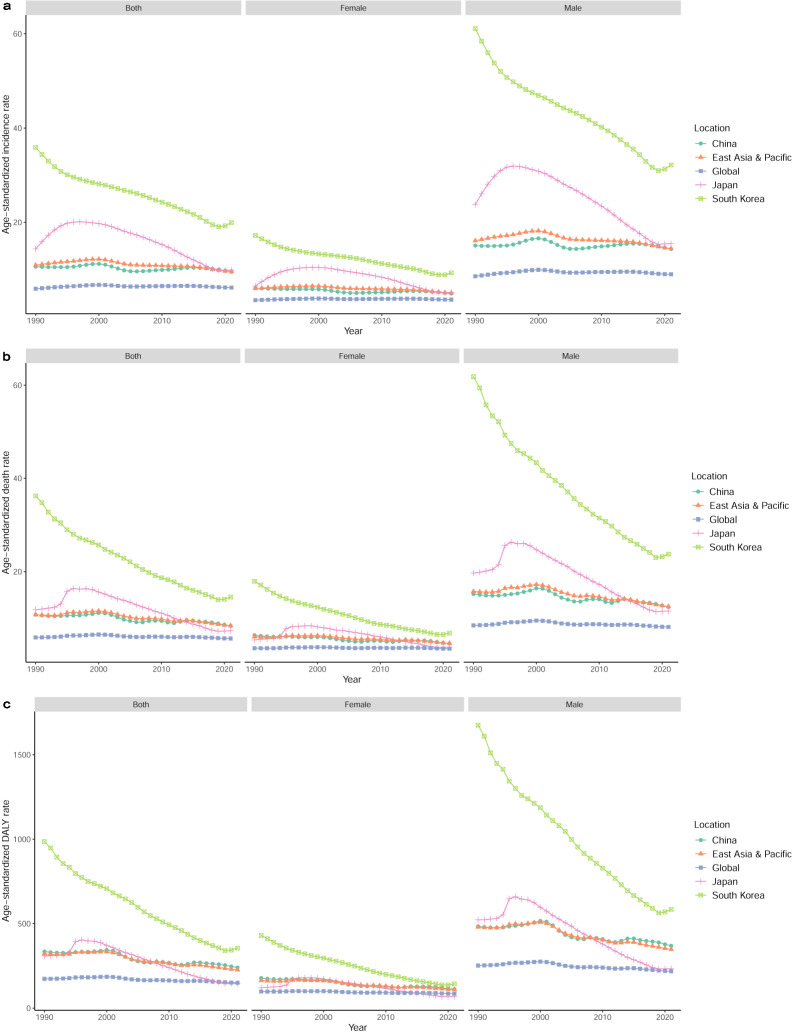
Trends for the ASIR (**a**), ASDR (**b**), and ASR of DALYs (**c**) of liver cancer in the world, East Asia & Pacific, China, Japan, and South Korea from 1990 to 2021. *ASIR* age-standardized incidence rate, *ASDR* age-standardized death rate, *ASR* age-standardized rate, *DALY* disability-adjusted life-year

**Fig. 2:**
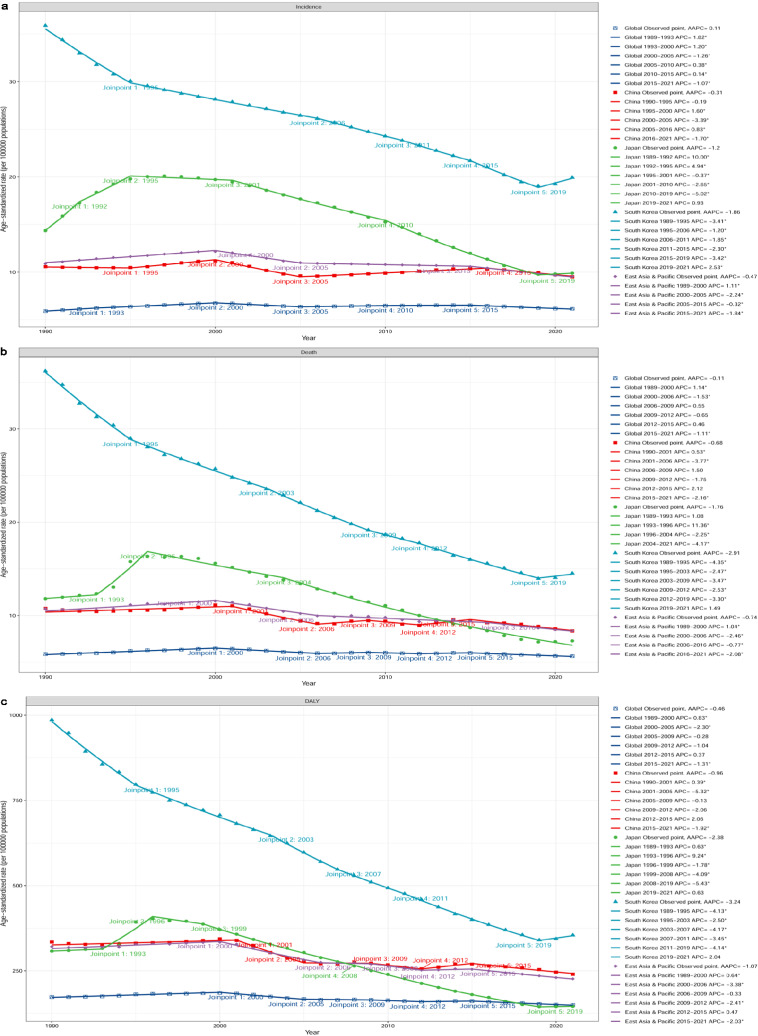
Annual percent changes for the ASIR, ASDR, and ASR of DALYs of liver cancer in the world, East Asia & Pacific, China, Japan, and South Korea from 1990 to 2021 calculated using Joinpoint regression analyses. *ASIR* age-standardized incidence rate, *ASDR* age-standardized death rate, *ASR* age-standardized rate, *DALY* disability-adjusted life-year

The ASIR, ASDR, and ASR of DALYs exhibited a decreasing trend in the three countries for both men and women between 1990 and 2021 (Table 2, Fig. 1). ASIR decreased the most in South Korea (− 44.44%) and the least in China (− 10.04%), similar to ASDR (− 59.87% in South Korea and − 22.35% in China) and ASR of DALYs (− 64.04% in South Korea and − 28.28% in China). From 1990 to 2021, both the ASIR and ASDR of LC exhibited an overall downward trend in China, with an AAPC of − 0.31% for ASIR and − 0.68% for ASDR (Fig. 2a, b). ASIR and ASDR decreased faster in Japan than in China (AAPC of − 1.20% for ASIR and − 1.76% for ASDR; Fig. 2a, b). In South Korea, the ASIR and ASDR decreased the fastest, with an AAPC of − 1.86% for ASIR and − 2.91% for ASDR (Fig. 2a, b).

### Cause distribution of LC burden in China, Japan, and South Korea

**Fig. 3:**
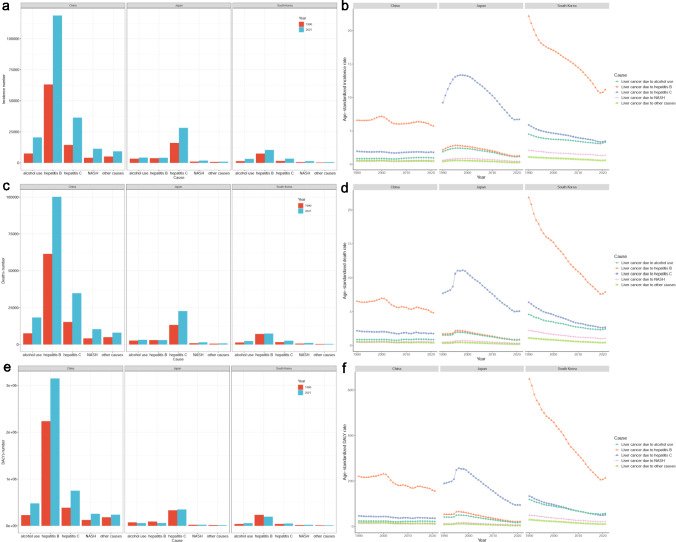
Age-standardized rate and number of five etiologies of liver cancer in China, Japan, and South Korea from 1990 to 2021. **a** incidence number of five etiologies of liver cancer in China, Japan, and South Korea; **b** age-standardized incidence rate of five etiologies of liver cancer in China, Japan, and South Korea; **c** deaths number of five etiologies of liver cancer in China, Japan, and South Korea; **d** age-standardized death rate of five etiologies of liver cancer in China, Japan, and South Korea; **e** DALYs number of five etiologies of liver cancer in China, Japan, and South Korea; **f** age-standardized DALY rate of five etiologies of liver cancer in China, Japan, and South Korea. *DALY* disability-adjusted life-year, *NASH* non-alcoholic steatohepatitis

**Fig. 4:**
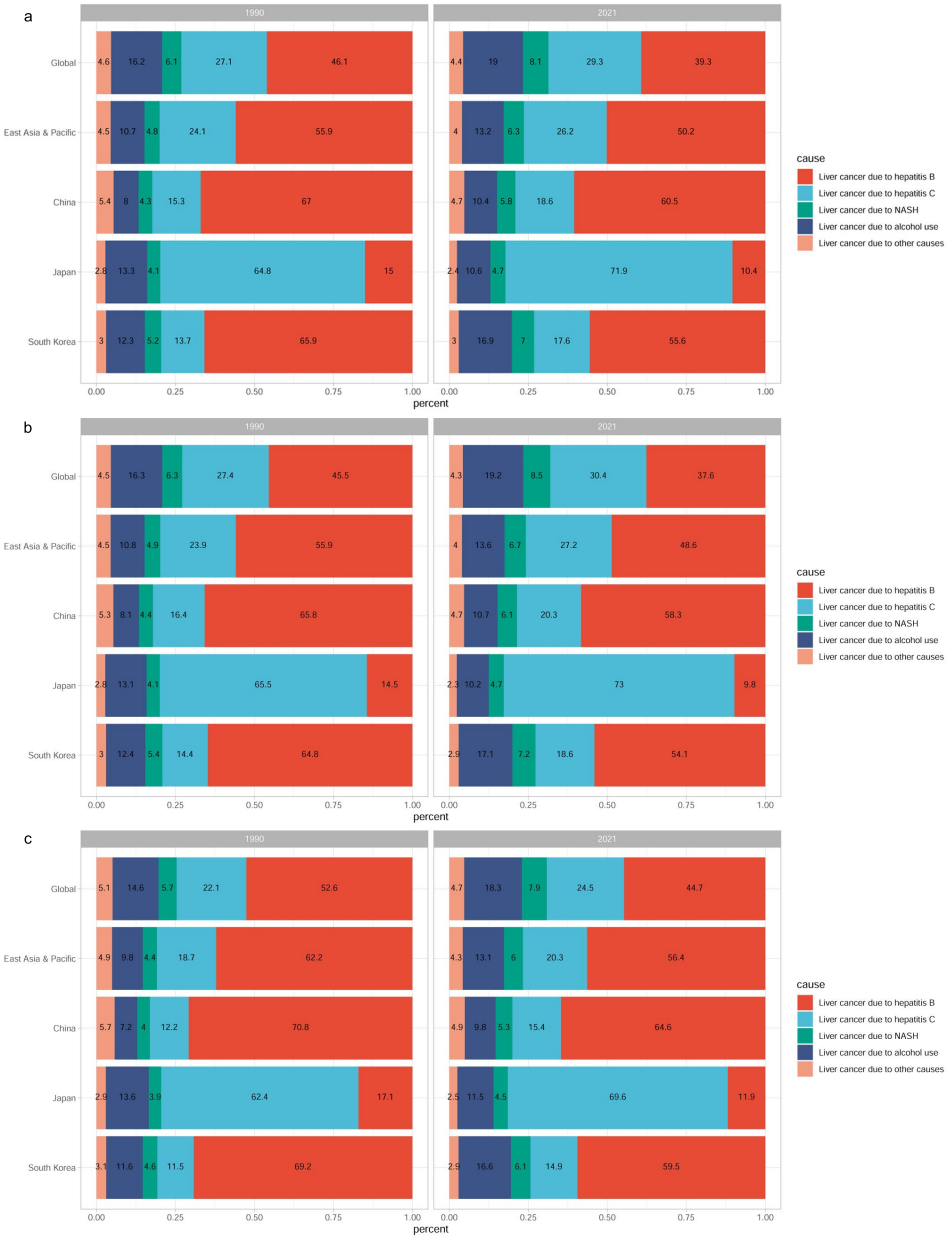
Cause distribution of liver cancer burden in China, Japan, and South Korea in 2021. **a** Incidence; **b** deaths; **c** DALYs. *DALY* disability-adjusted life-year, *NASH* non-alcoholic steatohepatitis

The ASR and number of five etiologies of LC and AAPCs of these ASRs in the three countries in 2021 are summarised in Table 3. Trends of ASR and number of five etiologies of LC in the three countries from 1990 to 2021 are presented in Fig. 3. During this period, the number of LC incidents and deaths due to the five etiologies increased. NASH was the highest-growing etiologies of incident LC cases (+ 178.36%) and deaths (+ 152.15%) in China (Figure S1). In contrast, alcohol use was the highest-growing etiologies of incidents (+ 126.41%) and deaths (+ 70.24%) in South Korea (Figure S1). In China, the ASIRs for HBV- and HCV-related LC decreased between 1990 and 2021 (AAPC of − 0.42% and − 0.25%, respectively), whereas the ASIRs for NASH- and alcohol-related LC increased (AAPC of 0.44% and 0.38%, respectively). In Japan and South Korea, the ASIRs, ASDRs, and ASR of DALYs for the five etiologies of LC decreased. The cause of LC varied greatly among the three countries (Fig. 4). In 2021, at the global level, HBV infections accounted for 39.3% of the total LC incident cases, followed by HCV infections (29.3%), alcohol use (19.0%), NASH (8.1%), and other causes (4.4%). HBV was responsible for 37.6% of LC mortality, HCV for 30.4%, alcohol use for 19.2%, NASH for 8.5%, and other causes for 4.3% worldwide (Fig. 4). HBV was the major cause of LC in China and South Korea. Notably, incidents and deaths from LC due to HBV were responsible for 60.5% and 58.3% of the total LC incidents and deaths, respectively, in China. The proportions of cases and deaths from LC due to HBV were 55.6% and 54.1%, respectively, in South Korea (Fig. 4). HCV was the second contributor to the LC burden in China and South Korea and the related incidents and deaths were approximately 15.0% of the total in these countries. HCV was the leading cause of incidents and deaths from LC in Japan, accounting for 71.9% and 73.0% of the total cases, respectively (Fig. 4). In contrast, HBV-related incidents and deaths only accounted for 10.4% and 9.8% of the total cases, respectively (Fig. 4).

**Table 3:** The ASR and number of five etiologies of liver cancer in the world, East Asia & Pacific, China, Japan, and South Korea in 2021, both sexes

Location	Etiologies	Incidence			Death			DALYs		
		Numbers	ASR	AAPC of ASR (95% CI)	Numbers	ASR	AAPC of ASR (95% CI)	Numbers	ASR	AAPC of ASR (95% CI)
World	Alcohol use	99,544 (80,957–120,402)	1.14 (0.93–1.38)	0.58 (0.55–0.61)	92,228 (75,053–112,160)	1.06 (0.86–1.29)	0.35 (0.22–0.48)	2,316,027 (1,887,013–2,845,789)	26.39 (21.53–32.28)	0.18 (0.05–0.32)
	Hepatitis B	206,366 (169,401–252,050)	2.37 (1.95–2.89)	− 0.22 (− 0.27 to − 0.17)	181,194 (148,896–221,685)	2.09 (1.72–2.55)	− 0.54 (− 0.81 to − 0.27)	5,668,199 (4,706,886–6,885,071)	65.36 (54.43–79.35)	− 0.79 (− 0.99 to − 0.59)
	Hepatitis C	154,062 (131,916–177,255)	1.82 (1.56–2.08)	0.26 (0.13–0.39)	146,522 (125,936–168,519)	1.74 (1.49–1.99)	0.05 (− 0.26 to 0.36)	3,098,870 (2,662,298–3,609,082)	35.85 (30.84–41.70)	− 0.31 (− 0.57 to − 0.05)
	NASH	42,291 (34,033–51,129)	0.49 (0.40–0.60)	0.99 (0.95–1.03)	40,925 (32,961–49,610)	0.48 (0.39–0.58)	0.82 (0.72–0.92)	995,475 (808,799–1,201,789)	11.50 (9.39–13.84)	0.60 (0.43–0.77)
	Other causes	22,892 (18,375–27,801)	0.27 (0.21–0.32)	0.09 (0.05–0.12)	20,590 (16,371–25,048)	0.24 (0.19–0.29)	− 0.17 (− 0.34 to − 0.01)	595,603 (484,797–732,313)	6.92 (5.64–8.48)	− 0.47 (− 0.68 to − 0.26)
East Asia & Pacific	Alcohol use	40,935 (31,695–53,231)	1.22 (0.95–1.57)	0.11 (0.04–0.17)	36,801 (28,494–47,433)	1.10 (0.85–1.41)	− 0.11 (− 0.46 to 0.24)	948,280 (725,857–1,253,448)	28.20 (21.71–37.00)	− 0.36 (− 0.60 to − 0.13)
	Hepatitis B	155,483 (125,220–194,323)	4.76 (3.85–5.94)	− 0.53 (− 0.59 to − 0.47)	131,945 (106,003–164,637)	4.04 (3.26–5.02)	− 1.01 (− 1.34 to − 0.68)	4,095,578 (3,299,029–5,161,486)	128.00 (103.68–161.04)	− 1.11 (− 1.48 to − 0.74)
	Hepatitis C	81,204 (68,137–94,742)	2.52 (2.13–2.93)	− 0.47 (− 0.63 to − 0.31)	73,887 (61,659–86,192)	2.32 (1.93–2.69)	− 0.73 (− 1.09 to − 0.37)	1,473,067 (1,227,755–1,758,158)	44.68 (37.28–53.09)	− 1.28 (− 1.62 to − 0.95)
	NASH	19,606 (15,372–24,230)	0.60 (0.47–0.74)	0.33 (0.29–0.38)	18,082 (14,226–22,534)	0.56 (0.44–0.69)	0.04 (− 0.22 to 0.30)	434,128 (336,003–539,612)	13.24 (10.32–16.29)	− 0.24 (− 0.52 to 0.03)
	Other causes	12,426 (9650–15,595)	0.38 (0.30–0.48)	− 0.60 (− 0.64 to − 0.56)	10,788 (8,349–13,498)	0.33 (0.26–0.41)	− 1.00 (− 1.39 to − 0.61)	309,637 (240,406–398,821)	9.75 (7.61–12.46)	− 1.22 (− 1.62 to − 0.81)
China	Alcohol use	20,464 (15,239–27,296)	0.94 (0.71–1.25)	0.38 (0.22–0.55)	18,317 (13,653–24,252)	0.85 (0.64–1.12)	0.01 (− 0.44 to 0.46)	477,847 (352,518–637,755)	22.01 (16.30–29.15)	− 0.25 (− 0.61 to 0.12)
	Hepatitis B	118,665 (92,280–153,556)	5.73 (4.48–7.38)	− 0.42 (− 0.51 to − 0.33)	100,194 (77,721–129,138)	4.83 (3.76–6.19)	− 0.92 (− 1.36 to − 0.49)	3,148,553 (2,442,865–4,109,014)	155.81 (121.32–201.99)	− 0.99 (− 1.46 to − 0.51)
	Hepatitis C	36,427 (28,404–44,840)	1.78 (1.41–2.18)	− 0.25 (− 0.39 to − 0.11)	34,899 (27,413–42,965)	1.74 (1.38–2.12)	− 0.58 (− 1.03 to − 0.13)	751,020 (585,482–933,695)	35.49 (27.67–44.05)	− 0.79 (− 1.29 to − 0.27)
	NASH	11,293 (8663–14,314)	0.54 (0.42–0.68)	0.44 (0.34–0.54)	10,409 (8,036–13,180)	0.51 (0.39–0.64)	0.13 (− 0.36 to 0.61)	256,209 (194,368–326,023)	12.22 (9.41–15.44)	− 0.23 (− 0.71 to 0.25)
	Other causes	9235 (7034–11,875)	0.45 (0.34–0.57)	− 0.56 (− 0.66 to − 0.46)	8,033 (6,102–10,231)	0.39 (0.30–0.49)	− 1.05 (− 1.56 to − 0.53)	237,416 (180,748–310,459)	11.82 (9.11–15.35)	− 1.15 (− 1.69 to − 0.61)
Japan	Alcohol use	4157 (3483–5023)	1.16 (0.98–1.38)	− 1.61 (− 1.73 to − 1.49)	3,162 (2,660–3,794)	0.83 (0.71–0.99)	− 1.98 (− 2.21 to − 1.76)	57,517 (48,684–69,158)	18.01 (15.37–21.30)	− 2.68 (− 2.87 to − 2.49)
	Hepatitis B	4065 (3223–4969)	1.27 (1.04–1.52)	− 1.73 (− 1.88 to − 1.58)	3,042 (2,393–3,718)	0.87 (0.71–1.05)	− 2.15 (− 2.37 to − 1.94)	59,409 (48,202–71,399)	21.93 (18.34–25.85)	− 2.83 (− 3.05 to − 2.60)
	Hepatitis C	28,136 (23,990–30,995)	6.72 (5.91–7.31)	− 1.02 (− 1.20 to − 0.84)	22,711 (19,030–24,914)	5.07 (4.40–5.51)	− 1.56 (− 1.99 to − 1.13)	348,478 (302,020–379,312)	94.43 (84.86–102.36)	− 2.22 (− 2.43 to − 2.00)
	NASH	1834 (1387–2272)	0.44 (0.35–0.53)	− 0.95 (− 1.20 to − 0.70)	1,470 (1,105–1,848)	0.33 (0.26–0.40)	− 1.28 (− 1.51 to − 1.05)	22,522 (17,796–27,361)	6.14 (5.01–7.31)	− 2.10 (− 2.33 to − 1.88)
	Other causes	945 (734–1156)	0.26 (0.21–0.31)	− 1.41 (− 1.63 to − 1.19)	731 (557–906)	0.18 (0.15–0.22)	− 1.82 (− 2.16 to − 1.48)	12,582 (10,154–15,150)	4.11 (3.43–4.83)	− 2.53 (− 2.85 to − 2.20)
South Korea	Alcohol use	3146 (2061–4659)	3.30 (2.17–4.84)	− 1.00 (− 1.04 to − 0.96)	2,334 (1,538–3,406)	2.44 (1.61–3.52)	− 2.02 (− 2.23 to − 1.81)	54,243 (35,350–81,191)	57.24 (37.83–84.62)	− 2.31 (− 2.54 to − 2.09)
	Hepatitis B	10,359 (8163–13,084)	11.18 (8.91–14.02)	− 2.19 (− 2.33 to − 2.06)	7,398 (5,806–9,454)	7.91 (6.22–10.05)	− 3.24 (− 3.45 to − 3.02)	193,930 (152,198–248,180)	213.57 (170.61–269.91)	− 3.51 (− 3.75 to − 3.27)
	Hepatitis C	3278 (2204–4717)	3.44 (2.33–4.89)	− 1.71 (− 1.79 to − 1.63)	2,549 (1,700–3,629)	2.70 (1.80–3.83)	− 2.76 (− 2.92 to − 2.59)	48,459 (32,204–70,331)	50.81 (34.11–73.11)	− 3.07 (− 3.24 to − 2.90)
	NASH	1297 (834–1978)	1.36 (0.88–2.06)	− 1.39 (− 1.44 to − 1.35)	990 (626–1,485)	1.04 (0.66–1.56)	− 2.44 (− 2.66 to − 2.22)	20,005 (12,991–30,308)	21.19 (13.88–31.66)	− 2.78 (− 3.01 to − 2.55)
	Other causes	552 (350–796)	0.59 (0.38–0.85)	− 1.95 (− 2.01 to − 1.89)	400 (249–579)	0.43 (0.27–0.61)	− 3.02 (− 3.23 to − 2.81)	9480 (6011–13,912)	10.42 (6.78–15.08)	− 3.35 (− 3.49 to − 3.21)

*ASR* age-standardized rate, *DALY* disability-adjusted life-year, *AAPC* average annual percentage change, *NASH* non-alcoholic steatohepatitis, *CI* confidence interval

### Sex and age distribution patterns in China, Japan, and South Korea, compared with those in the world and East Asia & Pacific region

Globally, almost 68.85% (364,355 [321,977–422,727]) of the incident cases occurred in men compared to 31.15% (164,848 [147,932–181,388]) in women in 2021 (Table 4); the male-to-female ratio for ASIR was 2.49. Men accounted for almost 67.10% (324,696 [288,483–376,834]) of the deaths and women for 32.90% (159,179 [142,936–175,021]), with a male-to-female ratio of 2.34 for ASDR. In the East Asia & Pacific area, men accounted for approximately 71.79% (222,918 [185,620–277,403]) of incident cases, while women made up 28.21% (87,618 [73,764–102,906]) of the cases in 2021; the male-to-female ratio was 2.86 for ASIR. In the same year and region, men accounted for approximately 70.28% (191,109 [158,834–238,726]) of the death cases, while women made up 29.72% (80,799 [67,807–94,978]), with a male-to-female ratio of 2.71 for ASDR. In China, 73.12% (143,788 [108,927–193,831]) of incident cases occurred in men compared to 26.88% (52,848 [41,045–67,026]) in women (Table 4), with a male-to-female ratio of 2.93 for ASIR. Men accounted for 71.20% (122,463 [93,115–164,816]) of all deaths, and women for 28.80% (49,605 [38,617–62,668]), with a male-to-female ratio of 2.71 for ASDR. Sex disparities in ASIR and ASDR were similar but larger in Japan and South Korea (ASIR in Japan: male-to-female ratio of 3.00; ASDR in Japan: 3.06; ASIR in South Korea: 3.46; ASDR in South Korea: 3.51).

**Table 4:** The ASR and number of liver cancer cases in the world, East Asia & Pacific, China, Japan, and South Korea in 2021, by sex

Variables	World		East Asia & Pacific		China		Japan		South Korea	
	Male	Female	Male	Female	Male	Female	Male	Female	Male	Female
Incidence rate (per 100,000)	8.98 (7.96–10.37)	3.60 (3.24–3.96)	14.42 (12.03–17.82)	5.05 (4.25–5.94)	14.34 (10.93–19.18)	4.89 (3.82–6.18)	15.48 (14.35–16.36)	5.16 (4.11–5.82)	32.16 (26.37–39.34)	9.29 (7.02–11.76)
Incidence numbers	364,355 (321,977–422,727)	164,848 (147,932–181,388)	222,918 (185,620–277,403)	87,618(73,764–102,906)	143,788 (108,927–193,831)	52,848 (41,045–67,026)	25,917 (23,708–27,594)	13,246 (9696–15,362)	13,768 (11,199–16,930)	4875 (3625–6196)
Death rate (per 100,000)	8.10 (7.24–9.37)	3.46 (3.11–3.80)	12.51 (10.43–15.50)	4.62 (3.88–5.44)	12.40 (9.46–16.55)	4.57 (3.57–5.76)	11.55 (10.67–12.10)	3.78 (2.97–4.24)	23.77 (19.67–29.24)	6.78 (5.16–8.51)
Death numbers	324,696 (288,483–376,834)	159,179(142,936–175,021)	191,109 (158,834–238,726)	80,799 (67,807–94,978)	122,463 (93,115–164,816)	49,605 (38,617–62,668)	20,243 (18,446–21,324)	10,879 (8022–12,568)	10,015 (8220–12,369)	3659 (2717–4616)
DALYs rate (per 100,000)	217.65 (191.58–255.35)	85.06 (77.25–93.70)	347.77 (286.45–439.91)	108.71 (91.21–129.98)	368.19 (279.67–490.95)	111.91 (87.16–141.96)	232.80 (219.42–242.54)	68.41 (57.70–74.64)	583.88 (478.97–712.66)	142.63 (113.69–176.14)
DALYs numbers	9,076,177 (7,970,743–10,670,814)	3,811,476 (3,455,663–4,199,822)	5,463,851 (4,473,188–6,942,544)	1,832,533 (1,535,991–2,186,959)	3,702,093 (2,805,347–4,985,654)	1,187,930 (924,053–1,513,173)	351,281 (326,745–367,518)	149,752 (116,034–169,178)	255,338 (208,125–312,026)	70,998 (55,829–87,946)

*ASR* age-standardized rate, *DALY* disability-adjusted life-year

ASIR and ASDR were markedly low in the 0–34-year age group but the indices rose significantly from 35 to 39 years and peaked in the 80+-year age group; the same trend was observed globally, in the East Asia & Pacific region, and the three countries (Figure S2). Globally, the incident cases, deaths, and DALYs of LC in older patients (≥ 60 years) accounted for 64.51%, 68.71%, and 50.73%, respectively, of the total burden (Table 5). In China, the incident cases, deaths, and DALYs of LC in the same population were 55.51%, 61.46%, and 43.67%, respectively, of the total burden (Table 5). The respective percentages were 93.27%, 95.12%, and 88.33% in Japan, and 70.37%, 75.32%, and 60.14% in South Korea (Table 5).

**Table 5:** The ASR and number of liver cancer cases in the world, East Asia & Pacific, China, Japan, and South Korea in 2021, by age group

Location	Age (years)	Incidence		Death		DALYs	
		Numbers	ASR	Numbers	ASR	Numbers	ASR
World	< 60	187,807 (162,785–220,360)	2.47 (2.14–2.90)	151,420 (131,603–177,227)	1.99 (1.73–2.33)	6,349,441 (5,504,688–7,449,489)	84.55 (73.22–99.30)
	60–80	261,492 (238,052–289,514)	28.05 (25.54–31.05)	244,797 (222,719–271,390)	26.29 (23.92–29.14)	5,563,172 (5,062,549–6,175,799)	595.37 (541.75–660.84)
	> 80	79,903 (66,358–88,992)	51.11 (42.39–56.95)	87,658 (73,386–97,382)	56.21 (46.98–62.48)	975,039 (821,599–1,081,432)	621.54 (522.93–689.65)
East Asia & Pacific	< 60	119,006 (96,070–149,343)	4.23 (3.41–5.32)	92,057 (74,289–116,019)	3.25 (2.62–4.10)	3,798,028 (3,061,464–4,793,749)	138.91 (111.85–175.50)
	60–80	143,236 (122,968–168,676)	39.42 (33.84–46.43)	129,560 (111,012–153,146)	35.65 (30.55–42.15)	2,941,692 (2,516,250–3,487,079)	809.80 (692.61–960.30)
	> 80	48,294 (39,391–55,514)	83.72 (68.14–96.26)	50,292 (41,007–57,969)	87.65 (71.29–101.04)	556,663 (457,143–641,808)	961.87 (788.14–1109.07)
China	< 60	87,488 (66,488–114,712)	4.92 (3.74–6.46)	66,317 (50,372–87,177)	3.69 (2.80–4.85)	2,754,595 (2,093,959–3,619,327)	160.43 (122.05–210.71)
	60–80	88,090 (70,306–109,123)	37.07 (29.58–45.95)	81,646 (65,383–101,225)	34.34 (27.49–42.58)	1,863,030 (1,488,027–2,318,671)	785.13 (626.84–977.71)
	> 80	21,058 (17,120–25,559)	64.04 (51.90–77.78)	24,105 (19,647–29,344)	74.27 (60.30–90.47)	272,397 (222,403–331,602)	821.55 (668.63–1000.47)
Japan	< 60	2635 (2389–2908)	1.79 (1.62–1.98)	1519 (1459–1582)	1.01 (0.97–1.05)	58,462 (56,140–60,887)	40.27 (38.60–42.00)
	60–80	18,236 (16,223–20,066)	50.74 (45.34–55.72)	13,260 (12,123–13,976)	36.25 (33.34–38.12)	269,425 (247,634–283,784)	780.15 (721.57–819.87)
	> 80	18,291 (13,958–21,223)	147.03 (113.20–170.12)	16,343 (12,644–18,510)	127.73 (99.99–143.89)	173,145 (135,325–195,430)	1398.57 (1104.30–1570.95)
South Korea	< 60	5,23 (3860–7784)	7.53 (5.24–10.66)	3375 (2340–4678)	4.52 (3.13–6.29)	130,069 (90,150–180,952)	179.66 (124.09–251.45)
	60–80	9547 (6903–12,724)	90.78 (65.65–120.99)	7109 (5115–9345)	67.83 (48.81–89.15)	160,139 (115,249–211,032)	1509.94 (1086.82–1989.39)
	> 80	3572 (2510–4876)	187.15 (131.17–255.62)	3190 (2270–4360)	169.17 (119.94–231.40)	36,128 (25,901–49,385)	1893.91 (1,353.84–2590.54)

*ASR* age-standardized rate, *DALY* disability-adjusted life-year

### Risk factors attributable to LC death and DALYs in the world, China, Japan, and South Korea

**Fig. 5:**
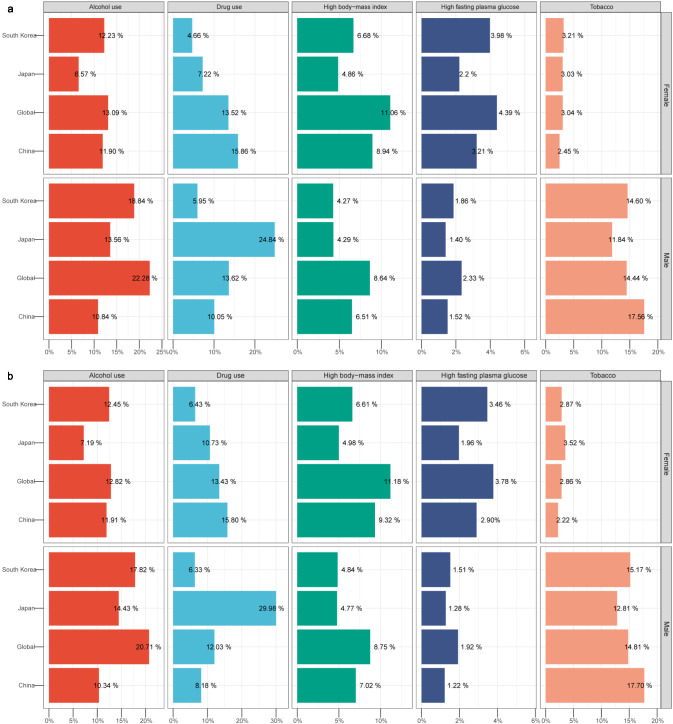
Proportion of liver cancer deaths and DALYs attributed to five risk factors in the world, East Asia & Pacific, China, Japan, and South Korea in 2021. **a** Deaths; **b** DALYs. *DALY* disability-adjusted life-year

The number of LC deaths and DALYs due to high body-mass index (BMI) increased globally and in the three countries from 1990 to 2021; the largest increase was noted in China (609.00% and 518.75%, respectively, Figure S3). The number of drug use-related deaths and DALYs for LC increased from 1990 to 2021 in the three countries and increased the highest in South Korea (244.52% for deaths and 173.25% for DALYs, Figure S3). From 1990 to 2021, the number of tobacco use-related DALYs and deaths increased in China and decreased in Japan and South Korea (Figure S3). The number of high alcohol use-related DALYs and deaths increased in China and South Korea, and the highest increases were recorded in China (124.72% for death and 90.87% for DALYs, Figure S3). The proportion of LC deaths attributed to risk factors varied by country and sex in 2021 (Fig. 5a). The largest percentage of tobacco-related LC deaths among men occurred in China (17.56%), higher than the global level (14.44%), whereas the lowest percentage was observed in Japan (11.84%). The percentage of high BMI-related LC deaths for men in China was higher than those in Japan and South Korea. The largest percentage of drug use-related LC deaths for men was observed in Japan (24.84%), also above the global level (13.62%), whereas the lowest was observed in South Korea (5.95%). South Korea exhibited the highest percentage of alcohol use-related LC deaths (18.84%). High fasting plasma glucose (FPG) was the lowest contributor to death in men globally and in the three countries. The highest percentage of high BMI-related LC deaths among women was observed in China (8.94%). The highest percentage of alcohol and drug use-related LC deaths among women was in South Korea (12.23%) and China (15.86%), respectively. High FPG and tobacco use were the lowest contributors to death in women worldwide and in the three countries.

The proportion of LC DALYs attributed to risk factors also varied by country and sex (Fig. 5b). Tobacco was the main contributor to DALYs in men in China; alcohol use was the primary contributor to DALYs in men and women in South Korea, and drug use was the primary contributor to DALYs in men in Japan. High FPG was the least significant contributor to DALYs worldwide and in the three countries.

### Predicted patterns and trends in LC burden from 2022 to 2046

**Fig. 6:**
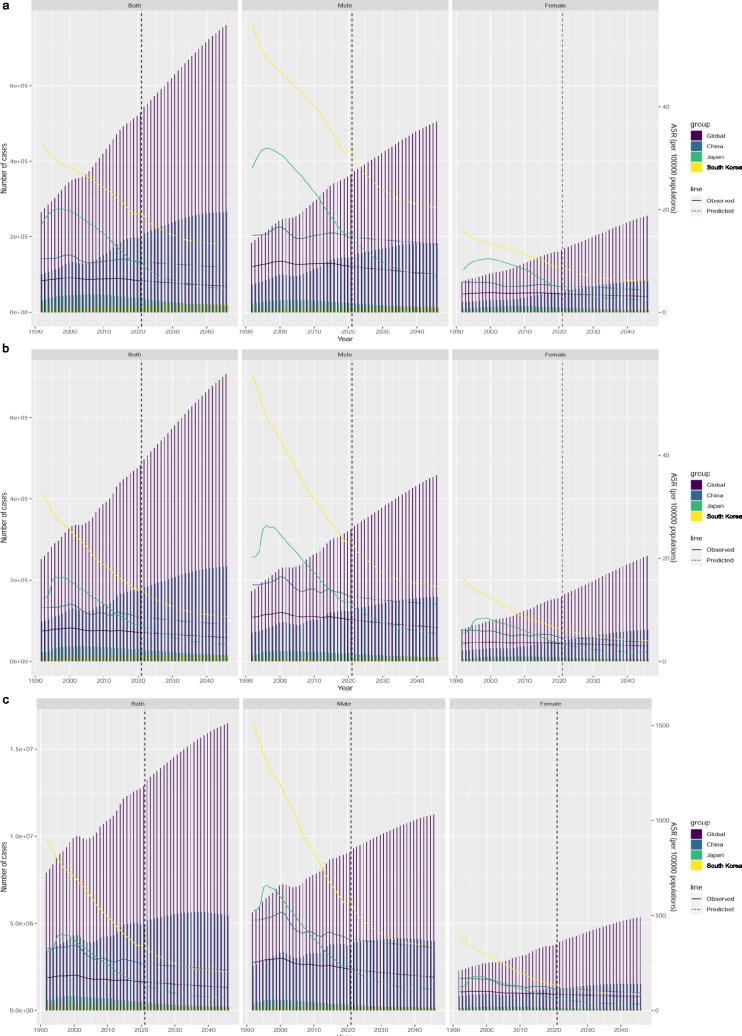
Predicted case numbers and age-standardized rate of liver cancer in the world, China, Japan, and South Korea from 2022 to 2046. **a** Incidence; **b** deaths; **c** DALYs

By 2046, the global LC burden was predicted to increase to 754,772 cases, 707,054 deaths, and 16,485,011 DALYs. Further, the predicted ASRs of incidence, death, and DALYs were 5.11, 4.68, and 122.80 per 100,000, respectively (Fig. 6). In China, the LC burden was predicted to increase to 260,235 cases, 233,447 deaths, and 5,443,299 DALYs. The ASRs of incidence, death, and DALYs were predicted to be 8.68, 7.29, and 211.61 per 100,000, respectively, by 2046. In Japan, the LC burden was predicted to increase to 16,854 cases, 14,483 deaths, and 201,088 DALYs; the ASRs of incidence, death, and DALYs were predicted to be 4.54, 3.51, and 72.11 per 100,000, respectively, by 2046. In South Korea, the LC burden was predicted to increase to 20,457 cases, 15,214 deaths, and 253,708 DALYs, and the ASRs of incidence, death, and DALYs were predicted to be 13.13, 9.20, and 213.52 per 100,000, respectively, by 2046. The burden of LC deaths and DALYs in 2046 is expected to be significantly greater in men than in women (Table 6).

**Table 6:** Prediction of the ASR and number of liver cancer cases in the world, China, Japan, and South Korea in 2046, by sex

Variables	World			China			Japan			South Korea		
	Male	Female	Male/female	Male	Female	Male/female	Male	Female	Male/female	Male	Female	Male/female
Incidence rate (per 100,000)	7.35	3.05	2.41	13.18	4.28	3.08	6.93	2.37	2.92	20.29	6.09	3.33
Incidence numbers	504,683	255,952	1.97	182,424	83,093	2.20	11,853	5434	2.18	15,448	5790	2.67
Death rate (per 100,000)	6.56	2.98	2.20	11.01	3.72	2.96	5.46	1.78	3.07	14.54	4.06	3.58
Death numbers	458,669	259,314	1.77	159,392	78,294	2.04	10,143	4929	2.06	11,882	4387	2.71
DALYs rate (per 100,000)	175.19	73.04	2.40	329.30	91.81	3.59	111.20	34.75	3.20	337.01	87.10	3.87
DALYs numbers	11,252,931	5,380,959	2.09	3,968,912	1,518,912	2.61	148,870	61,677	2.41	204,824	62,988	3.25

*ASR* age-standardized rate, *DALY* disability-adjusted life-year

## Discussion

LC is a health concern that must be addressed in China, Japan, and South Korea. Our research comprehensively evaluated the current LC burden and temporal trends in these countries and assessed the possible risk factors. These findings can provide scientific references for policy makers in these countries to formulate effective prevention and intervention strategies.

In 2021, LC incident cases, mortality, and DALYs were highest in China and lowest in South Korea. South Korea recorded the highest ASRs of incidence, mortality, and DALYs, while Japan the lowest. From 1990 to 2021, LC incidence and mortality in the three countries increased. The decomposition analysis revealed that the increase was largely due to ageing. This finding is consistent with Li’s research [15]. Older populations are at increased risk of developing LC [16]. According to a previous study, LC incidence increases sharply after 40 years of age [17]. In this study, we also found that ASIR and ASDR increased sharply after 35–39 years of age. Of note, our study demonstrated that LC incidence and mortality were more pronounced in older than in younger patients in the three East Asian countries. In Japan, the proportion of older individuals (≥ 60 years) with LC was the highest among all LC populations in the three countries. Japan is a super-aged society, and the proportion of persons aged ≥ 65 years increased from 4.9% in 1950 to 28.4% in 2019. According to our results, LC burden is heavier in the older population in Japan. Ageing of the global population, together with an increased frequency of exposure to risk factors linked to the formation of LC, such as NASH and obesity, imply that the incidence of LC in older adults will continue to grow in the future. Epidemiological change (early detection, diagnosis and treatment, and reducing potential morbidity) could partially offset the contribution of ageing to the increased incidents and deaths due to LC. Thus, more attention should be directed towards comprehensive measures for LC prevention, control, and treatment strategies in this age group.

The ASRs for LC tended to decrease from 1990 to 2021. The ASIR, ASDR, and ASR of DALYs declined the least and slowest in China and the most and fastest in South Korea. The AAPCs for South Korea and Japan were higher than those for China, suggesting more effective cancer prevention and control approaches in the former countries. Consistent with previous findings [18], HBV and HCV remain the primary causes of LC burden in the three countries. More than 60% of LC incidents and deaths are attributable to HBV and HCV infections, suggesting that LC is largely preventable if risk factor control. In our study, we found that the ASIR and ASDR from LC due to HBV in China and South Korea have significantly decreased between 1990 and 2021. This is likely due to the initiation of a series of HBV vaccination programs in these countries. China has already met the national target of reducing HBsAg prevalence to < 1% among children younger than 5 years and has averted an estimated 16–20 million HBV carriers through baby hepatitis B immunisation [19]. South Korea has also implemented a nationwide HBV vaccine and perinatal transmission prevention program. Infant immunisation coverage reached 98%, whereas the HBsAg-positivity rate among those aged 10–18 years decreased to 0.1–0.2% [20]. The only country in Asia with predominantly HCV-related LC, rather than HBV-related, is Japan, although the ASIR and ASDR from LC due to HCV infection in this country decreased significantly between 1990 and 2021. Additionally, HBV-related incidents, deaths, and DALYs in Japan exhibited the lowest increases from 1990 to 2021, and the corresponding ASIR and ASDR decreased significantly. Although there is presently no effective vaccine to prevent new or re-infection with HCV, Japan has enacted several national plans to protect against HCV infection. The national and local governments share the expenses of screening for HBV and HCV in residents over the age of 40 years [21]. As a result, the estimated number of patients with HBV or HCV hepatitis in Japan is decreasing [22]. More effective strategies against HCV infection are needed to meet the goals set by the World Health Organization (WHO).

Alcohol use and NASH were the third and fourth primary causes of LC, respectively, which were also preventable. NASH was the highest-growing etiologies for incident LC cases and deaths in China, and the corresponding ASIR and ASDR increased; this is consistent with previous findings [23]. According to a previous study, NASH is driven by metabolic syndrome and is associated with obesity and insulin resistance [24]. The prevalence of NASH has been found to increase significantly in developed countries, such as the United States and Canada [25].The Western lifestyle is more prone to develop NASH. As China's economy has expanded, the country's economically developed regions have seen a steady transition from an Eastern to Western eating pattern, which may explain the increase in ASIR and ASDR of LC due to NASH. The global burden of NASH parallels the increase in obesity rates [26]. Our study also observed that high BMI was a primary risk factor with the greatest increase in prevalence in China from 1990 to 2021. High BMI is indicative of obesity, and numerous epidemiological studies have demonstrated that obesity is not only a risk factor for NASH but also a recognised independent risk factor for LC. Recently, obesity and excess weight have become more common phenomena, affecting over one-third of the global population. China has the largest population with obesity in the world, and experiences a growth rate of 0.32% per year [27]. Fortunately, we found that the ASIR and ASDR of LC due to NASH decreased in Japan and South Korea. According to a recent study, Japan and South Korea have low obesity rates, which may lead to a significant difference in the burden of LC due to NASH between Japan, South Korea, and China [25]. In China, obesity and potential metabolic risk factors need to be managed effectively to restrict the rising burden of NASH-related LC. Weight loss should be promoted for patients with NASH, and formal weight loss programs should be considered in patients at high risk of NASH-related LC. Maintaining a healthy lifestyle would decrease the incidence of metabolic diseases and obesity-related liver impairment, thereby lowering the burden of NASH-related LC. Alcohol use was the main contributor to the LC DALYs in men and women in South Korea. Over the past four decades, alcohol consumption has become an increasingly common lifestyle practice in South Korea [28]. According to a recent WHO report, South Korea has the largest per capita alcohol consumption among Asian countries [29]. This finding highlights the need for policies aimed at reducing harmful alcohol use. Policies such as raising the price and taxes for alcohol may reduce the burden of alcohol-related LC in South Korea.

Additional risk factors for developing LC include smoking, high FPG, and drug use. Smoking is a common lifestyle risk factor for LC. Our study indicated that smoking was responsible for the high mortality and DALYs of LC in China, Japan, and South Korea, which is strongly related to the notable smoking prevalence in these countries. In Japan, drug use was the main lifestyle risk factor for LC. The increasing prevalence of these risk factors may drive future changes in the LC burden. Considering the increasing trend, effective preventative measures targeting these risk factors are needed. China, Japan, and South Korea should develop personalized cancer prevention measures based on their respective national situations and different risk factors profiles.

Our results confirmed that the LC incidence and mortality are higher in men than in women in the three East Asian countries. Women experience a relatively lower burden of LC, which may be related to a better lifestyle (less alcohol intake and tobacco smoking, low levels of obesity, and other factors) and a significantly lower prevalence of HBV infection. Our analysis generated prediction patterns between 2022 and 2046 and demonstrated larger increases in LC incidence and mortality in women than in men, thus suggesting that this disparity might close in the near future.

Our study had some limitations. Firstly, estimation of incidence, deaths, and DALYs that is somewhat biased due to disparities in the coverage and quality of cancer registration and reporting systems across the three countries. For example, the cancer registration and reporting system in China only covers approximately 33% of the population and the registered data is not comprehensive enough, whereas the system in Japan covers approximately 70% of cancer cases [30]. Secondly, the GBD 2021 study lacks data regarding the histological subcategories of LC, such as hepatocellular carcinoma and cholangiocarcinoma. Further research is required to identify the burden of each histological group in China, Japan, and South Korea. Lastly, data regarding the tumor size or stages of LC incident cases are essential, and future studies investigating LC stages in China, Japan, and South Korea and the percentages of early- and late-stage LC are required.

## Conclusions

LC remains a significant disease burden in China, Japan, and South Korea now and in the next 25 years. HBV and HCV infections are still the primary causes of LC in these countries. Regarding etiologies and risk factors, NASH and high BMI in China, alcohol use in South Korea, and drug use in Japan should receive greater attention. More efficient preventive strategies should be designed to promote a healthy lifestyle and control LC.

## Supplementary Information

Below is the link to the electronic supplementary material.

**Figure S1.** Relative changes in the numbers and age-standardized rates for incidents, deaths, and DALYs of liver cancer by five etiologies between 1990 and 2021 in China, Japan, and South Korea. *DALY* disability-adjusted life-year, *NASH* non-alcoholic steatohepatitis (PDF 900 KB)

**Figure S2.** Age-specific rates for incidence, death, and DALY of liver cancer. *DALY* disability-adjusted life-year (PDF 1034 KB)

**Figure S3.** Trends in the percentages of liver cancer deaths and DALYs attributed to five risk factors in the world, China, Japan, and South Korea from 1990 to 2021. **a** Deaths; **b** DALYs. **c** Relative changes in the percentages of liver cancer deaths and DALYs attributed to risk factors between 1990 and 2021. *DALY* disability-adjusted life-year (PDF 3493 KB)

Supplementary file4 (DOCX 26 KB)

## Data Availability

The data used for the analyses are publicly available from the Global Health Data Exchange (GHDx) query tool (https://vizhub.healthdata.org/gbd-results/).

## Abbreviations

AAPC: Average annual percentage change
APC: Annual percentage change
ASDR: Age-standardized death rate
ASIR: Age-standardized incidence rate
ASR: Age-standardized rate
BMI: Body mass index
CI: Confidence interval
DALY: Disability-adjusted life-year
FPG: Fasting plasma glucose
GBD: Global Burden of Disease
GHDx: Global Health Data Exchange
HBV: Hepatitis B virus
HCV: Hepatitis C virus
LC: Liver cancer
NASH: Non-alcoholic steatohepatitis
UI: Uncertainty interval
WHO: World Health Organization

## References

[1] Bray F, Laversanne M, Sung H, Ferlay J, Siegel RL, Soerjomataram I, et al. Global cancer statistics 2022: GLOBOCAN estimates of incidence and mortality worldwide for 36 cancers in 185 countries. CA Cancer J Clin. 2024;74(3):229–263 38572751 10.3322/caac.21834

[2] Gomaa AI, Khan SA, Toledano MB, Waked I, Taylor-Robinson SD. Hepatocellular carcinoma: epidemiology, risk factors and pathogenesis. World J Gastroenterol. 2008;14(27):4300–4308 18666317 10.3748/wjg.14.4300 PMC2731180

[3] Choi S, Kim BK, Yon DK, Lee SW, Lee HG, Chang HH, et al. Global burden of primary liver cancer and its association with underlying aetiologies, sociodemographic status, and sex differences from 1990–2019: a DALY-based analysis of the Global Burden of Disease 2019 study. Clin Mol Hepatol. 2023;29(2):433–452 36597018 10.3350/cmh.2022.0316 PMC10121317

[4] McGlynn KA, Petrick JL, El-Serag HB. Epidemiology of hepatocellular carcinoma. Hepatology (Baltimore, MD). 2021;73(Suppl 1):4–13 32319693 10.1002/hep.31288 PMC7577946

[5] Sohn W, Lee HW, Lee S, Lim JH, Lee MW, Park CH, et al. Obesity and the risk of primary liver cancer: a systematic review and meta-analysis. Clin Mol Hepatol. 2021;27(1):157–174 33238333 10.3350/cmh.2020.0176 PMC7820201

[6] Ho NT, Abe SK, Rahman MS, Islam R, Saito E, Gupta PC, et al. Diabetes is associated with increased liver cancer incidence and mortality in adults: a report from Asia Cohort Consortium. Int J Cancer. 2024;155(5):854–870 38661292 10.1002/ijc.34965

[7] Ko KP, Shin A, Cho S, Park SK, Yoo KY. Environmental contributions to gastrointestinal and liver cancer in the Asia-Pacific region. J Gastroenterol Hepatol. 2018;33(1):111–120 28960448 10.1111/jgh.14005

[8] Sarin SK, Kumar M, Eslam M, George J, Al Mahtab M, Akbar SMF, et al. Liver diseases in the Asia-Pacific region: a Lancet Gastroenterology & Hepatology Commission. Lancet Gastroenterol Hepatol. 2020;5(2):167–228 31852635 10.1016/S2468-1253(19)30342-5 PMC7164809

[9] Schumacher AE, Kyu HH, Aali A, Abbafati C, Abbas J, Abbasgholizadeh R, et al. Global age-sex-specific mortality, life expectancy, and population estimates in 204 countries and territories and 811 subnational locations, 1950-2021, and the impact of the COVID-19 pandemic: a comprehensive demographic analysis for the Global Burden of Disease Study 2021. Lancet (London, England). 2024;403(10440):1989–2056 38484753 10.1016/S0140-6736(24)00476-8 PMC11126395

[10] Murray CJ, Aravkin AY, Zheng P, Abbafati C, Abbas KM, Abbasi-Kangevari M, et al. Global burden of 87 risk factors in 204 countries and territories, 1990–2019: a systematic analysis for the Global Burden of Disease Study 2019. Lancet (London, England). 2020;396(10258):1223–1249 33069327 10.1016/S0140-6736(20)30752-2 PMC7566194

[11] Vos T, Lim SS, Abbafati C, Abbas KM, Abbasi M, Abbasifard M, et al. Global burden of 369 diseases and injuries in 204 countries and territories, 1990–2019: a systematic analysis for the Global Burden of Disease Study 2019. Lancet (London, England). 2020;396(10258):1204–1222 33069326 10.1016/S0140-6736(20)30925-9 PMC7567026

[12] Das GP. Standardization and decomposition of rates: a user’s manual. Washington, DC: US Department of Commerce Economics and Statistics Administration; 1993

[13] Tapper EB, Parikh ND. Mortality due to cirrhosis and liver cancer in the United States, 1999–2016: observational study. BMJ (Clin Res Ed). 2018;362:k2817 10.1136/bmj.k2817 PMC6050518 30021785

[14] Møller B, Fekjaer H, Hakulinen T, Sigvaldason H, Storm HH, Talbäck M, et al. Prediction of cancer incidence in the Nordic countries: empirical comparison of different approaches. Stat Med. 2003;22(17):2751–2766 12939784 10.1002/sim.1481

[15] Li M, Hu M, Jiang L, Pei J, Zhu C. Trends in cancer incidence and potential associated factors in China. JAMA Netw Open. 2024;7(10):e2440381 39432306 10.1001/jamanetworkopen.2024.40381 PMC11581522

[16] Macias RIR, Monte MJ, Serrano MA, González-Santiago JM, Martín-Arribas I, Simão AL, et al. Impact of aging on primary liver cancer: epidemiology, pathogenesis and therapeutics. Aging (Albany NY). 2021;13(19):23416–23434 34633987 10.18632/aging.203620 PMC8544321

[17] Yuen MF, Hou JL, Chutaputti A. Hepatocellular carcinoma in the Asia pacific region. J Gastroenterol Hepatol. 2009;24(3):346–353 19220670 10.1111/j.1440-1746.2009.05784.x

[18] Akinyemiju T, Abera S, Ahmed M, Alam N, Alemayohu MA, Allen C, et al. The burden of primary liver cancer and underlying etiologies from 1990 to 2015 at the global, regional, and national level: results from the global burden of disease study 2015. JAMA Oncol. 2017;3(12):1683–1691 28983565 10.1001/jamaoncol.2017.3055 PMC5824275

[19] Liang X, Bi S, Yang W, Wang L, Cui G, Cui F, et al. Epidemiological serosurvey of hepatitis B in China—declining HBV prevalence due to hepatitis B vaccination. Vaccine. 2009;27(47):6550–6557 19729084 10.1016/j.vaccine.2009.08.048

[20] Lee CH, Choi GH, Choi HY, Han S, Jang ES, Chon YE, et al. Core indicators related to the elimination of hepatitis B and C virus infection in South Korea: a nationwide study. Clin Mol Hepatol. 2023;29(3):779–793 37188331 10.3350/cmh.2023.0110 PMC10366799

[21] Kanto T. Messages from Japan policy for viral hepatitis. Glob Health Med. 2021;3(5):249–252 34782865 10.35772/ghm.2021.01078 PMC8562090

[22] Tanaka J, Akita T, Ohisa M, Sakamune K, Ko K, Uchida S, et al. Trends in the total numbers of HBV and HCV carriers in Japan from 2000 to 2011. J Viral Hepatitis. 2018;25(4):363–372 10.1111/jvh.12828 29193549

[23] Huang DQ, Singal AG, Kono Y, Tan DJH, El-Serag HB, Loomba R. Changing global epidemiology of liver cancer from 2010 to 2019: NASH is the fastest growing cause of liver cancer. Cell Metab. 2022;34(7):969–77.e2 35793659 10.1016/j.cmet.2022.05.003 PMC9762323

[24] Loomba R, Sanyal AJ. The global NAFLD epidemic. Nat Rev Gastroenterol Hepatol. 2013;10(11):686–690 24042449 10.1038/nrgastro.2013.171

[25] Liu C, Zhu S, Zhang J, Wu P, Wang X, Du S, et al. Global, regional, and national burden of liver cancer due to non-alcoholic steatohepatitis, 1990–2019: a decomposition and age-period-cohort analysis. J Gastroenterol. 2023;58(12):1222–1236 37665532 10.1007/s00535-023-02040-4

[26] Quek J, Chan KE, Wong ZY, Tan C, Tan B, Lim WH, et al. Global prevalence of non-alcoholic fatty liver disease and non-alcoholic steatohepatitis in the overweight and obese population: a systematic review and meta-analysis. Lancet Gastroenterol Hepatol. 2023;8(1):20–30 36400097 10.1016/S2468-1253(22)00317-X

[27] Wang Y, Zhao L, Gao L, Pan A, Xue H. Health policy and public health implications of obesity in China. Lancet Diabetes Endocrinol. 2021;9(7):446–461 34097869 10.1016/S2213-8587(21)00118-2

[28] Jang JY, Kim DJ. Epidemiology of alcoholic liver disease in Korea. Clin Mol Hepatol. 2018;24(2):93–99 29544241 10.3350/cmh.2017.0079 PMC6038943

[29] World Health Organization. Global status report on alcohol and health 2018. Geneva: World Health Organization; 2018

[30] Matsuda T, Sobue T. Recent trends in population-based cancer registries in Japan: the Act on Promotion of Cancer Registries and drastic changes in the historical registry. Int J Clin Oncol. 2015;20(1):11–20 25351534 10.1007/s10147-014-0765-4

