# Supplementary figures and images for: Burden, trends, and predictions of liver cancer in China, Japan, and South Korea: analysis based on the Global Burden of Disease Study 2021
Source: Hepatol Int. 2025 Jan 11;19(2):441–59. doi: 10.1007/s12072-024-10763-6 (PMC12003535; doi:10.1007/s12072-024-10763-6)

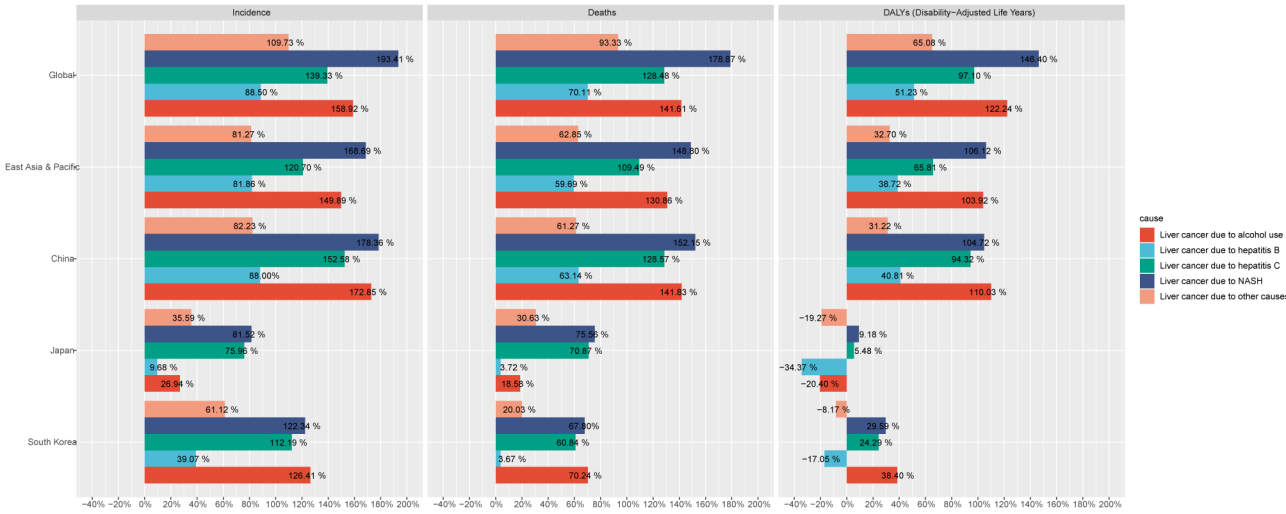

Supplement: Supplementary file 1 — Figure S1. Relative changes in the numbers and age-standardized rates for incidents, deaths, and DALYs of liver cancer by five etiologies between 1990 and 2021 in China, Japan, and South Korea. DALY disability-adjusted life-year, NASH non-alcoholic steatohepatitis (PDF 900 KB) [file 12072_2024_10763_MOESM1_ESM.pdf]

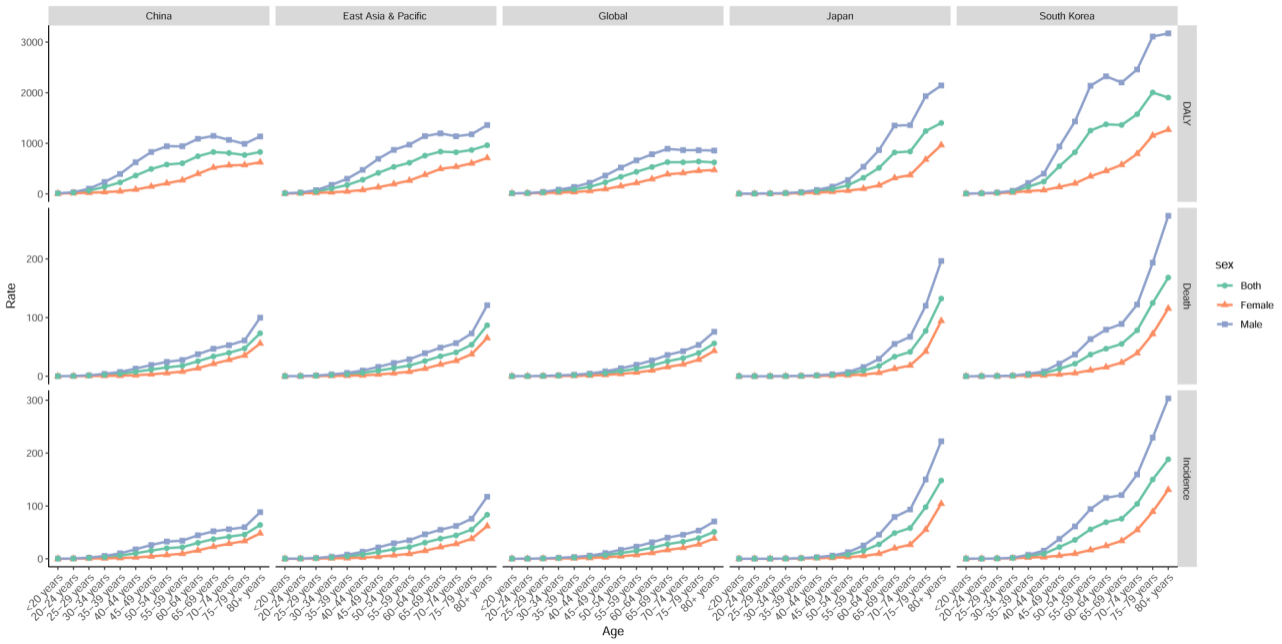

Supplement: Supplementary file 2 — Figure S2. Age-specific rates for incidence, death, and DALY of liver cancer. DALY disability-adjusted life-year (PDF 1034 KB) [file 12072_2024_10763_MOESM2_ESM.pdf]

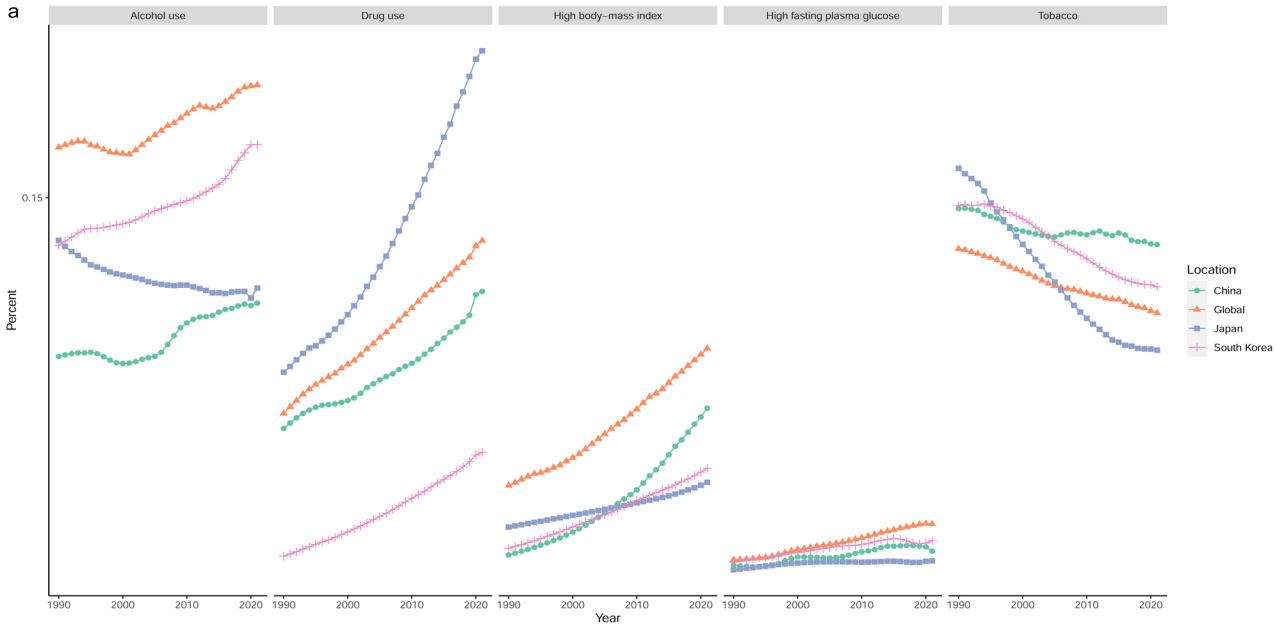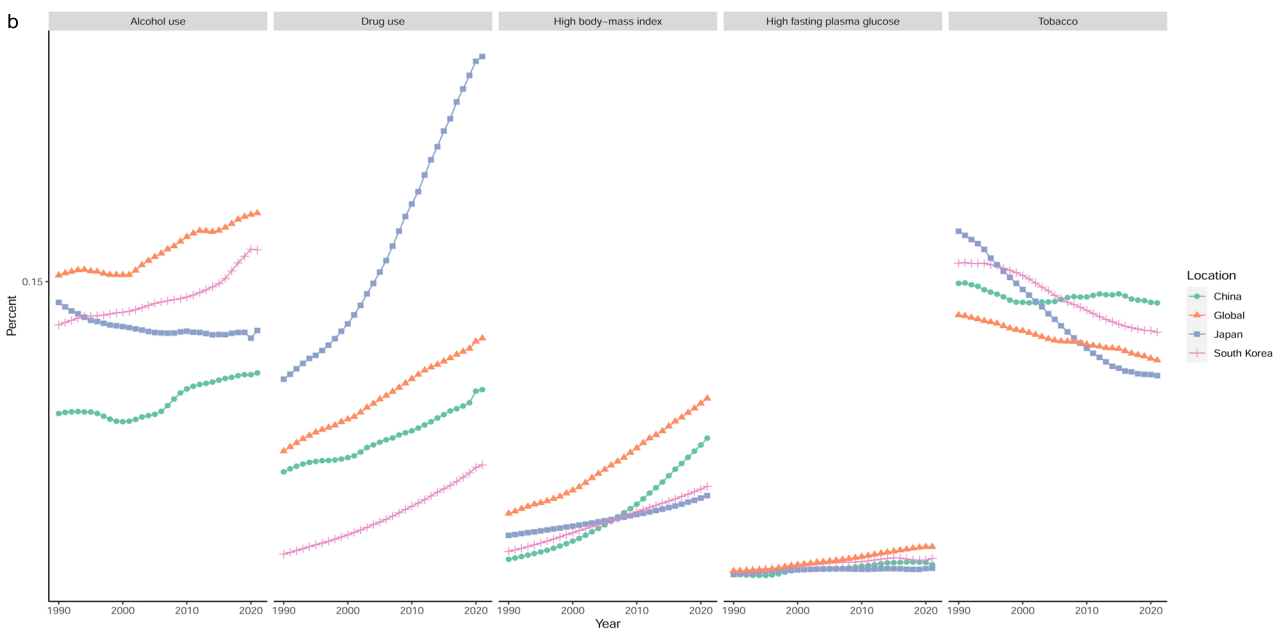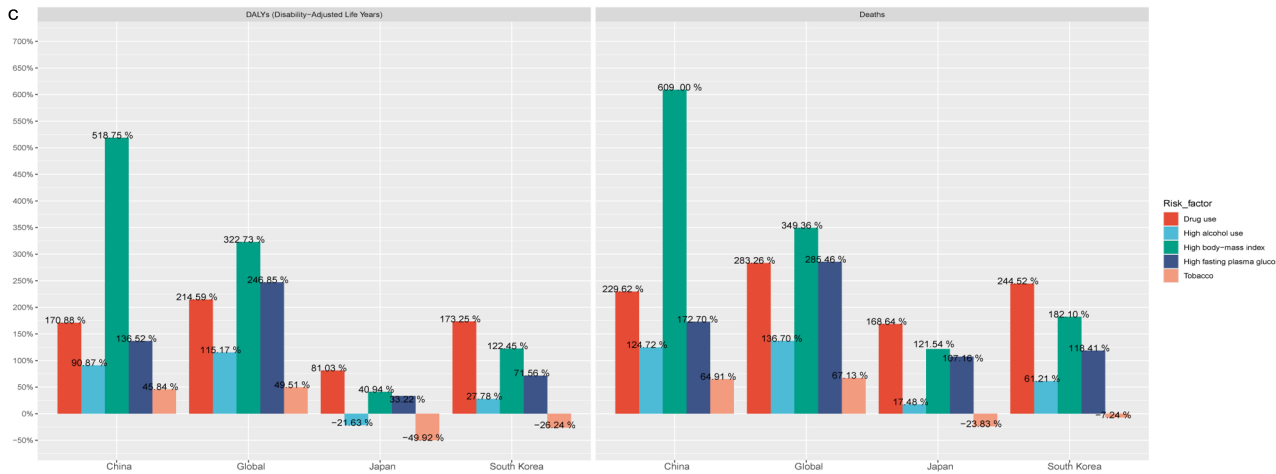

Supplement: Supplementary file 3 — Figure S3. Trends in the percentages of liver cancer deaths and DALYs attributed to five risk factors in the world, China, Japan, and South Korea from 1990 to 2021. a Deaths; b DALYs. c Relative changes in the percentages of liver cancer deaths and DALYs attributed to risk factors between 1990 and 2021. DALY disability-adjusted life-year (PDF 3493 KB) [file 12072_2024_10763_MOESM3_ESM.pdf]
