# Supplementary material for: Burden, trends, and predictions of liver cancer in China, Japan, and South Korea: analysis based on the Global Burden of Disease Study 2021
Source: Hepatol Int. 2025 Jan 11;19(2):441–59. doi: 10.1007/s12072-024-10763-6 (PMC12003535; doi:10.1007/s12072-024-10763-6)
Supplement: Supplementary file 4 — Supplementary file4 (DOCX 26 KB) [file 12072_2024_10763_MOESM4_ESM.docx]

**Table S1. Changes in incidents, deaths, and DALYs of liver cancer according to population-level determinants including ageing, population growth and epidemiological change from 1990 to 2021.**

| **Location** | **Sex** | **Measure** | **Overll difference** | **Ageing** | **Population** | **Epidemiological change** | **Percent change of ageing,**  **%** | **Percent change of population,**  **%** | **Percent change of epidemiological change,**  **%** | **Overall change,**  **%** |
| --- | --- | --- | --- | --- | --- | --- | --- | --- | --- | --- |
| China | Both | Incidence | 100,202 | 89,832 | 27,314 | -16,944 | 93.2 | 28.3 | -17.6 | 103.9 |
|  |  | Deaths | 77,131 | 88,453 | 25,423 | -36,745 | 93.2 | 26.8 | -38.7 | 81.2 |
|  |  | DALYs | 1,595,159 | 2,191,164 | 785,572 | -1,381,577 | 66.5 | 23.8 | -41.9 | 48.4 |
|  | Female | Incidence | 26,623 | 27,166 | 7,756 | -8,299 | 103.6 | 29.6 | -31.6 | 101.5 |
|  |  | Deaths | 22,972 | 28,215 | 7,624 | -12,866 | 105.9 | 28.6 | -48.3 | 86.3 |
|  |  | DALYs | 355,218 | 608,358 | 204,163 | -457,303 | 73.1 | 24.5 | -54.9 | 42.7 |
|  | Male | Incidence | 73,579 | 62,157 | 19,053 | -7,630 | 88.5 | 27.1 | -10.9 | 104.8 |
|  |  | Deaths | 54,159 | 59,933 | 17,360 | -23,133 | 87.7 | 25.4 | -33.9 | 79.3 |
|  |  | DALYs | 1,239,940 | 1,556,023 | 564,706 | -880,789 | 63.2 | 22.9 | -35.8 | 50.4 |
| East Asia & Pacific | Both | Incidence | 154,600 | 135,064 | 55,392 | -35,855 | 86.6 | 35.5 | -23.0 | 99.1 |
|  |  | Deaths | 122,048 | 129,795 | 50,957 | -58,704 | 86.6 | 34.0 | -39.2 | 81.4 |
|  |  | DALYs | 2,358,514 | 3,084,816 | 1,500,066 | -2,226,369 | 62.5 | 30.4 | -45.1 | 47.8 |
|  | Female | Incidence | 45,403 | 40,841 | 15,607 | -11,045 | 96.7 | 37.0 | -26.2 | 107.6 |
|  |  | Deaths | 39,073 | 41,042 | 14,964 | -16,933 | 98.4 | 35.9 | -40.6 | 93.6 |
|  |  | DALYs | 595,455 | 836,097 | 382,271 | -622,912 | 67.6 | 30.9 | -50.4 | 48.1 |
|  | Male | Incidence | 109,197 | 94,792 | 39,443 | -25,038 | 83.4 | 34.7 | -22.0 | 96.0 |
|  |  | Deaths | 82,975 | 89,479 | 35,703 | -42,208 | 82.7 | 33.0 | -39.0 | 76.7 |
|  |  | DALYs | 1,763,059 | 2,246,395 | 1,106,695 | -1,590,031 | 60.7 | 29.9 | -43.0 | 47.6 |
| Global | Both | Incidence | 284,513 | 132,462 | 143,486 | 8,565 | 54.1 | 58.6 | 3.5 | 116.3 |
|  |  | Deaths | 244,906 | 130,310 | 135,463 | -20,867 | 54.5 | 56.7 | -8.7 | 102.5 |
|  |  | DALYs | 5,333,986 | 3,032,812 | 3,909,350 | -1,608,177 | 40.2 | 51.8 | -21.3 | 70.6 |
|  | Female | Incidence | 89,153 | 40,758 | 44,950 | 3,445 | 53.8 | 59.4 | 4.6 | 117.8 |
|  |  | Deaths | 83,052 | 42,117 | 44,292 | -3,357 | 55.3 | 58.2 | -4.4 | 109.1 |
|  |  | DALYs | 1,600,462 | 859,189 | 1,159,230 | -417,957 | 38.9 | 52.4 | -18.9 | 72.4 |
|  | Male | Incidence | 195,360 | 94,083 | 98,048 | 3,230 | 55.7 | 58.0 | 1.9 | 115.6 |
|  |  | Deaths | 161,854 | 90,726 | 90,762 | -19,634 | 55.7 | 55.7 | -12.1 | 99.4 |
|  |  | DALYs | 3,733,524 | 2,202,587 | 2,735,350 | -1,204,412 | 41.2 | 51.2 | -22.5 | 69.9 |
| Japan | Both | Incidence | 14,426 | 19,905 | 459 | -5,938 | 80.5 | 1.9 | -24.0 | 58.3 |
|  |  | Deaths | 10,815 | 17,692 | 373 | -7,250 | 87.1 | 1.8 | -35.7 | 53.3 |
|  |  | DALYs | -30,087 | 254,489 | 7,639 | -292,216 | 47.9 | 1.4 | -55.0 | -5.7 |
|  | Female | Incidence | 6,956 | 7,035 | 220 | -299 | 111.8 | 3.5 | -4.7 | 110.6 |
|  |  | Deaths | 5,665 | 6,470 | 183 | -988 | 124.1 | 3.5 | -18.9 | 108.6 |
|  |  | DALYs | 35,507 | 81,716 | 3,080 | -49,289 | 71.5 | 2.7 | -43.1 | 31.1 |
|  | Male | Incidence | 7,470 | 14,137 | 125 | -6,792 | 76.6 | 0.7 | -36.8 | 40.5 |
|  |  | Deaths | 5,150 | 12,301 | 101 | -7,252 | 81.5 | 0.7 | -48.0 | 34.1 |
|  |  | DALYs | -65,595 | 194,844 | 2,233 | -262,672 | 46.7 | 0.5 | -63.0 | -15.7 |
| South Korea | Both | Incidence | 7,293 | 14,685 | 2,460 | -9,852 | 129.4 | 21.7 | -86.8 | 64.3 |
|  |  | Deaths | 2,632 | 13,696 | 2,168 | -13,232 | 124.0 | 19.6 | -119.8 | 23.8 |
|  |  | DALYs | -13,738 | 314,687 | 58,467 | -386,892 | 92.5 | 17.2 | -113.8 | -4.0 |
|  | Female | Incidence | 1,997 | 3,968 | 641 | -2,612 | 137.9 | 22.3 | -90.8 | 69.4 |
|  |  | Deaths | 760 | 3,864 | 585 | -3,689 | 133.3 | 20.2 | -127.2 | 26.2 |
|  |  | DALYs | -5,935 | 73,325 | 13,234 | -92,494 | 95.3 | 17.2 | -120.2 | -7.7 |
|  | Male | Incidence | 5,296 | 11,880 | 1,856 | -8,440 | 140.2 | 21.9 | -99.6 | 62.5 |
|  |  | Deaths | 1,872 | 11,008 | 1,626 | -10,763 | 135.2 | 20.0 | -132.2 | 23.0 |
|  |  | DALYs | -7,803 | 267,685 | 46,143 | -321,631 | 101.7 | 17.5 | -122.2 | -3.0 |

**DALY=disability-adjusted life-year.**
